# Gender differences in adverse event reports associated with antidiabetic drugs

**DOI:** 10.1038/s41598-020-74000-4

**Published:** 2020-10-16

**Authors:** Kyung-In Joung, Gyu-Won Jung, Han-Heui Park, Hyesung Lee, So-Hee Park, Ju-Young Shin

**Affiliations:** School of Pharmacy, Sungkyunkwan University, 2066 Seobu-ro, Jangan-gu, Suwon, Gyeong gi-do South Korea

**Keywords:** Health care, Medical research

## Abstract

Little is known about gender-specific reporting of adverse events (AEs) associated with antidiabetic drugs. This study was to assess the gender-related difference in AEs reporting associated with antidiabetic agents. The number of antidiabetic drug-AE pairs associated was identified using the Korea Adverse Event Reporting System database. Prevalence of diabetes was estimated using the Health Insurance Review and Assessment Service-National Patients Sample database. Reporting rate per 10,000 people was calculated by dividing drug-AE pairs with the number of antidiabetic drug users by gender. Gender difference was presented with risk ratio (reporting rate ratio) of women to men. Antidiabetic agent-associated AEs were more frequently reported by women than men throughout body organs and drug classes. 13 out of 17 system organ class level disorders with significant gender differences were reported more often by women than men. By drug class, gender-specific reporting rates were observed in most of the drug classes, especially in newer classes such as glucagon-like peptide-1 analog (GLP1-RA), sodium glucose co-transporter-2 inhibitor (SGLT2i), and thiazolidinedione (TZD). Looking into preferred term level for each drug class, women dominated the reports of class-specific AEs of newer antidiabetic drugs such as urinary tract/genital infection (all reported by women) in SGLT2i, edema in TZD (risk ratio (RR) 12.56), and hyperglycemia in insulin users (RR 15.35). Gender differences in antidiabetic-associated AE reporting often attributed to women. Explanations for these different report levels by gender should be further investigated.

## Introduction

Diabetes is one of the critical health problems with a risk of developing serious life-threatening complications and increased mortality. The global prevalence of diabetes has been increasing that the affected people were expected to be 693 million by 2045 worldwide^1^. The burden of diabetes is also raising in Korea and a recent study estimated that 14% of Korean adults suffer from the disease^2^. Since life-long therapies with glucose-lowering agents are unavoidable in most diabetic patients, optimal drug choice through recognizing drug adverse events (AEs) and ensuring compliance with medications are key to glycemic control.

Sex differences in physiological, hormonal, and genetic conditions that affect pharmacokinetics and pharmacodynamics of a drug may be the reason for unequal occurrence of drug AEs between women and men^3–5^. Some studies have overviewed gender differences in adverse drug events reporting and found that women report AEs more often than men^6–8^. Several other pieces of researches focused on the specific drug or drug class to look more closely at gender differences in AE reporting. They found that the report prevalence was higher in women than in men. However, discrepancies in report rate by gender varied according to the event categories or drug subclass^9,10^.

Regarding antidiabetic drugs, studies on the gender difference in AEs are rare, and even in clinical trials, the impact of gender was not usually assessed. Meanwhile, unfavorable results for women such as a more adverse cardiovascular disease risk profile, a higher likelihood of failing treatment goals, and more reported AEs after metformin treatment, than men, were occasionally demonstrated^11–14^. While two studies encompass the entire range of drugs to assess gender differences in AE reporting, they were not focused on antidiabetic class^7,8^. This study aimed to assess the gender-related difference in reported AEs among antidiabetics users.

## Methods

### Data sources

Two nationwide-databases were used: one is for AE reports, and the other is for measuring the size of antidiabetic exposure. The number of AE reports containing antidiabetic drugs was identified using the 2016 Korea Adverse Event Reporting System (KAERS) database, and the prevalence of diabetes in 2016 was estimated using the Health Insurance Review and Assessment Service-National Patients Sample (HIRA-NPS) database.

The KAERS is a computerized AE reporting system developed by the Korea Institute of Drug Safety and Risk Management (KIDS) in 2012. It contains the voluntary AE reports from healthcare professionals, consumers, regional pharmacovigilance centers, and market authorization holders including pharmaceutical companies, but also includes AE reports based on post-marketing surveillance, observation studies such as pharmacoepidemiologic studies to collect safety information of drug products, and other drug adverse reaction surveillance program. The KAERS database is aligned with the international standards and WHO-UMC (Uppsala Monitoring Centre) international drug monitoring program. To date, the KAERS database contains more than 1 million reports and 228,939 reports were newly integrated into the database in 2016. Each report includes information on administered drugs, AEs, patients, and reporters. Serious AE is defined as any AE occurring the following outcomes and coded in the KAERS database: death, a life-threatening experience, inpatient hospitalization or prolongation of existing hospitalization, a persistent or significant disability or incapacity, a congenital disability, or any other significant medical event requiring intervention. KAERS uses the Anatomical Therapeutic Chemical (ATC) classification to code drug names, and the World Health Organization-Adverse Reaction Terminology (WHO-ART) to code AEs and medication errors^15^.

The claims data of HIRA is national data compiled from healthcare providers across the country that corresponds to the number of claims submitted by patients. South Korea has a universal health coverage system that the National Health Insurance covers approximately 98% of the entire Korean population that the claims data of HIRA encompass about 90% of the Korean population. The HIRA-NPS data is a stratified random sample of the HIRA data with representativeness, comprising 3% of all patients per year. The data contains information on medical utilization including demographics, diagnosis, treatment, procedures, and prescription drugs which provide a valuable resource for healthcare service research. HIRA-NPS uses 8-digit variables to code drug name and the International Classification of Diseases, 10^th^ edition (ICD-10) from the World Health Organization^16^.

### Outcome and exposure definition

The outcome was an occurrence of AE associated with antidiabetic drugs and the number of antidiabetic drug-AE combination in AE reports was calculated to be an estimation. The source database in KAERS from January 1, 2016, to December 31, 2016, included 228,939 reports and the reports corresponded to 735,370 drug-AE pairs after combining all of the events and drugs from each report. Among them, only pairs on antidiabetic drugs were included. Inclusion criteria for antidiabetic drugs was those recommended as standards of medical care for diabetes as follows: insulin, biguanides, sulfonylureas, alpha-glucosidase inhibitors (AGI), thiazolidinediones (TZD), dipeptidyl peptidase 4 inhibitors (DPP4i), glucagon-like peptide-1 analog (GLP-1RA), and SGLT2i, and other oral glucose-lowering drugs. After excluding pairs without information of gender and sulfonamide-AE pairs (only two pairs were reported), 15,669 antidiabetic drug-event combinations were finally included.

To estimate the exposed population, we used the HIRA-NPS database as the KAERS database lacks denominator information. The total number of the population exposed to antidiabetic drugs was defined as those diagnosed with diabetes mellitus (DM) (ICD10: E10-E14) at least once during 2016. We also extracted the number of patients according to the prescribed antidiabetic class. A person who was once prescribed for each class during 2016 was defined as a patient exposed to the corresponding drug class. As the HIRA-NPS data was 3% sampled, the number of diabetic patients in the entire Korean population was calculated by multiplying the identified numbers from HIRA-NPS by 100/3.

### Identification of gender difference

Gender differences in the occurring antidiabetic drugs associated AEs were assessed by comparing the reporting rates of the drug-event combinations by gender. The reporting rates were computed as the number of reported drug-event combinations divided by the population exposed to corresponding drugs, expressed as rate per 10,000 persons summarized at the System Organ Class (SOC) level and drug class according to the ATC classification system. The reporting rate of the individual event at preferred term (PT) level was derived for each drug class and shown in Table 4 only when the difference in reporting rate between the genders is statistically significant and substantial: i.e., arbitrarily more than three times the difference. For these drug-AE combinations, we additionally calculated the reporting odds ratio (ROR) to assess the disparity in the signal generation. The ROR compares the ratio of case/non-case for a drug of interest with the corresponding ratio for all other drugs^17^.

### Statistical analyses

The demographic characteristics of diabetic patients and the basic information of the event reports were described by gender. DM patients were analyzed according to age group, DM type, coexisting conditions, and concomitant drugs. For AE reports, the distribution of age group, seriousness of events, report type, and report source was featured. We calculated the differences in the frequencies between women and men to compare the distribution of the analyzed variables. The risk of women to men was estimated as reporting rate ratio with the 95% confidence interval (CI). The criteria for a statistically significant difference in the reporting rate between genders were determined as risk ratio not equal to 1, and 95% CI not including 1. Statistical analyses were performed using SAS 9.4 software (SAS Institute Inc., Cary, NC, USA) and Excel 2010 (Microsoft Corp., Redmond, WA, USA).

## Results

### Descriptive data

In total, 115,048 diabetic patients were identified in the HIRA-NPS database during 2016 with 61,089 (46.9%) men and 53,959 (53.1%) women, which were converted to 2,036,300 men and 1,798,633 women in the total Korean population. Women with diabetes have a higher percentage of older adults than men, while the proportion of older adults in their 40s and 50s is significantly higher in women than in men. All comorbidities examined except nephropathy were more prevalent in men than in women, and hypertension, dyslipidemia, and cardiovascular disease were among the highest comorbid conditions in both genders in this order. Comedications with antihypertensives (except ACEi/ARB) and statin were more frequent in men than in women as well. Generally, the patterns of comorbidities and comedications seemed not to be particularly gender-specific. This background information is presented in Supplementary Table S1.

Basic information on antidiabetic drugs-related AE reports was presented in Table 1. We identified 7200 and 8469 antidiabetic drug-event pairs for men and women respectively from the KAERS database during 2016. Overall reporting rate per 10,000 people calculated by dividing drug-AE pairs with the number of antidiabetic drug users was higher in women than men (35 pairs in men vs. 47 pairs in women). On the contrary, the serious events were more frequently reported by men than women (14.8 pairs in men vs. 13.6 pairs in women). Both spontaneous reports and survey research accounted for the majority of the report types for both genders, while women presented higher occupancy of spontaneous report type compared to men (55.2% vs. 44.8%). By reporter, both reports from doctors and consumers accounted for more than 80% of the total diabetic drug-event pairs reported in both men and women. While drug-event pairs in men were reported most frequently by doctors (47.8%), whereas those in women were most frequently reported by consumers (43.6%). Notably, during the study period, most of the AE reports relevant to antidiabetic drugs were reported by pharmaceutical companies in Korea, which was quite different from the general feature of the national reporting: this seems to be due to the undergoing re-examination, an active surveillance by the Korean Ministry of Food and Drug Safety. 46 out of 72 antidiabetic drugs were re-examined over that time (Table 2).

**Table 1:** Distribution of antidiabetic drug-AE pairs extracted from KAERS database between January 1, 2016 and December 31, 2016.

Categories	Women (N = 8469)		Men (N = 7200)		Difference in percentage
	Drug-AE pairs	%	Drug-AE pairs	%	
**Age, years (n, %)**					
≤ 39	760	(9.0)	319	(4.4)	4.6
40–49	802	(9.5)	769	(10.7)	− 1.2
50–59	1468	(17.3)	1576	(21.9)	− 4.6
60–69	2145	(25.3)	1754	(24.4)	0.9
70–79	1969	(23.2)	1614	(22.4)	0.8
≥ 80	470	(5.5)	379	(5.3)	0.2
Missing	855	(10.1)	789	(11.0)	− 0.9
**Serious adverse events (n, %)**					
Yes	2451	(28.9)	2654	(36.9)	− 8.0
No	6018	(71.1)	4546	(63.1)	8.0
**Report type (n, %)**					
Spontaneous	4675	(55.2)	3226	(44.8)	10.4
Survey research	2570	(30.3)	2970	(41.3)	− 11.0
Literature	33	(0.4)	22	(0.3)	0.1
Etc	1191	(14.1)	982	(13.6)	0.5
**Report source by person (n, %)**					
Doctor	3247	(38.3)	3442	(47.8)	− 9.5
Pharmacist	1012	(11.9)	668	(9.3)	2.6
Nurse	346	(4.1)	243	(3.4)	0.7
Consumer	3696	(43.6)	2718	(37.8)	5.8
Other	99	(1.2)	68	(0.9)	0.3
Missing	69	(0.8)	61	(0.8)	0.0
**Report source by affiliation (n, %)**					
Regional pharmacovigilance center	1975	(23.3)	1366	(19.0)	4.3
Pharmaceutical company	6460	(76.3)	5801	(80.6)	− 4.3
Medical institution	26	(0.3)	12	(0.2)	0.1
Consumer	1	(0.0)	0	(0.0)	0.0
Other	7	(0.1)	21	(0.3)	− 0.2

*KAERS* Korea Adverse Event Reporting System, *AE* adverse event.

**Table 2:** Frequencies of drug-AE pairs, reporting rate by gender, and its ratio of women to men, summarized at SOC level.

SOC	Women		Men		Risk ratio^b^ (95% CI)
	Drug-AE pairs	Reporting rate^a^	Drug-AE pairs	Reporting rate^a^	
Overall	11,269	62.65	9905	48.64	1.29 (1.25–1.32)
Foetal disorders	194	1.08	2	0.01	109.82 (27.27–442.25)
Reproductive disorders	134	0.75	34	0.17	4.46 (3.06–6.50)
Special senses other, disorders	14	0.08	6	0.03	2.64 (1.02–6.87)
Hearing and vestibular disorders	46	0.26	20	0.10	2.60 (1.54–4.40)
Neoplasms	126	0.70	85	0.42	1.68 (1.27–2.21)
Application site disorders	296	1.65	206	1.01	1.63 (1.36–1.94)
Gastro-intestinal system disorders	2132	11.85	1538	7.55	1.57 (1.47–1.68)
Psychiatric disorders	531	2.95	384	1.89	1.57 (1.37–1.79)
Heart rate and rhythm disorders	112	0.62	91	0.45	1.39 (1.06–1.84)
Skin and appendages disorders	596	3.31	488	2.40	1.38 (1.23–1.56)
Musculo-skeletal system disorders	389	2.16	319	1.57	1.36 (1.19–1.6)
Metabolic and nutritional disorders	2343	13.03	1945	9.55	1.36 (1.28–1.45)
Central and peripheral nervous system disorders	927	5.15	818	4.02	1.28 (1.17–1.41)
Vision disorders	156	0.87	149	0.73	1.19 (0.95–1.48)
Resistance mechanism disorders	113	0.63	108	0.53	1.18 (0.91–1.54)
White cell and RES* disorders	146	0.81	142	0.70	1.16 (0.92–1.47)
Urinary system disorders	446	2.48	452	2.22	1.12 (0.98–1.27)
Body as a whole—general disorders	1060	5.89	1123	5.51	1.07 (0.98–1.16)
Secondary terms—events	370	2.06	394	1.93	1.06 (0.92–1.23)
Vascular (extracardiac) disorders	101	0.56	123	0.60	0.93 (0.71–1.21)
Respiratory system disorders	550	3.06	670	3.29	0.99 (0.83–1.04)
Endocrine disorders	19	0.11	25	0.12	0.86 (0.47–1.56)
Red blood cell disorders	52	0.29	78	0.38	0.75 (0.53–1.07)
Platelet, bleeding and clotting disorders	66	0.37	107	0.53	0.70 (0.51–0.95)
Cardiovascular disorders, general	117	0.65	192	0.94	0.69 (0.55–0.87)
Myo-, endo-, pericardial and valve disorders	52	0.29	86	0.42	0.68 (0.49–0.97)
Liver and biliary system disorders	176	0.98	319	1.57	0.62 (0.52–0.75)
Neonatal and infancy disorders	2	0.01	0	0	–
Collagen disorders	1	0.00	1	0	–
Poison specific terms	2	0.01	0	0	–

*SOC* system organ class, *AE* adverse event, *CI* confidence interval.

^a^Reporting rate per 10,000 people was calculated over the total number of diabetes patients (women = 1,798,633; men = 2,036,300).

^b^Risk ratio = reporting rate of women/reporting rate of men.

### Gender differences in reporting at the SOC level

**Figure 1:**
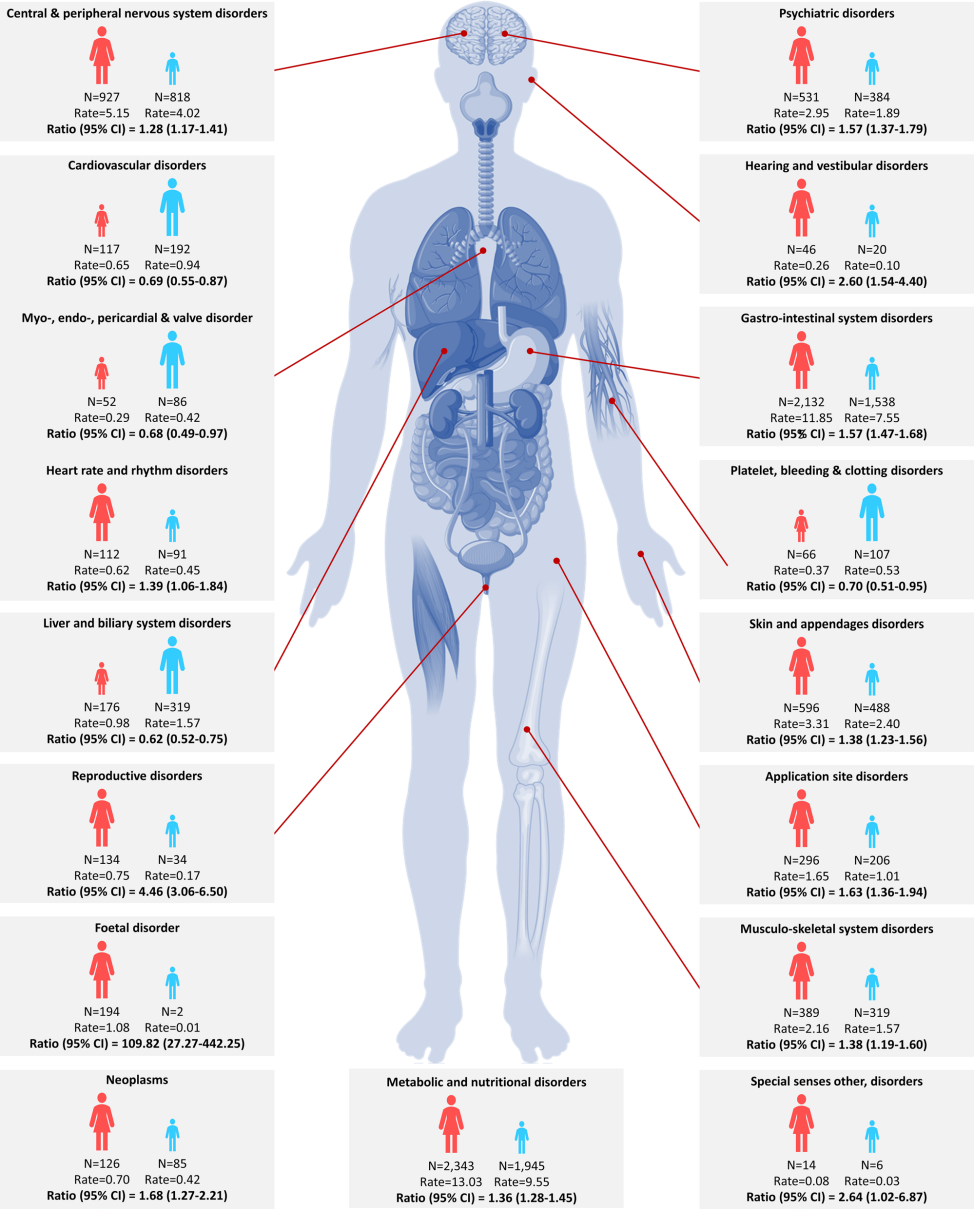
Disorders at SOCs level that demonstrated a significant difference between men and women. Rate, reporting rate per 10,000 people; Ratio, reporting rate ratio of women to men. The human body image in this figure was sourced from VectorStock, Royalty Free Vectors. https://www.vectorstock.com/royalty-free-vector/diagram-of-organs-of-the-human-body-vector-9717196 Accessed April 2019.

Frequencies of drug-AE pairs, reporting rate per 10,000 patients by gender, and its ratio of women to men were summarized at SOC level (Table 2). Both men and women had the highest reporting rates of metabolic and nutritional disorders, followed by gastrointestinal system disorders. In a wide range of disorders at the SOC level, women reported higher drug-event reporting rates than men. Of the 30 SOC level disorders, gender differences were detected in 17 SOCs. 13 of these indicated higher reporting rates in women than men. Disorders at SOCs levels that demonstrated a significant difference between the genders were illustrated in Fig. 1.

### Gender differences in the reporting by antidiabetic drug class

Examination of the antidiabetic drug-event pairs by drug class showed particularly high rates of GLP-1RA-, insulin-, and SGLT2i- related reporting in both men and women. Higher reporting rates in women were also observed throughout the analysis by drug class. Of the 9 classes, 7 classes had significantly higher reporting rates in women than in men. In particular, newer classes such as GLP-1RA and SGLT2i were relatively small in the number of patients treated, but the related drug-event reporting was quite frequent in men and women, and even the largest gender differences (Table 3).

**Table 3:** Frequencies of drug-AE pairs and reporting rate according to the drug class by gender, and its ratio of women to men.

Drug class	Women (N = 8469)			Men (N = 7200)			Risk ratio^b^ (95% CI)
	Number of drug user	Drug-AE pairs	Reporting rate^a^	Number of drug user	Drug-AE pairs	Reporting rate^a^	
GLP-1RA	3167	193	609.5	2800	73	260.7	2.34 (1.79–3.05)
SGLT2 inhibitors	72,400	482	66.6	78,767	233	29.6	2.25 (1.93–2.63)
Thiazolidinedione	145,500	221	15.2	203,033	196	9.7	1.57 (1.30–1.91)
Insulin	245,800	4919	200.1	281,267	3901	138.7	1.44 (1.38–1.50)
Sulfonylurea	660,133	1096	16.6	834,033	1090	13.1	1.27 (1.17–1.38)
Metformin	1,210,667	2530	20.9	1,482,000	2445	16.5	1.27 (1.20–1.34)
DPP-4 inhibitors	812,667	1493	18.4	1,045,533	1602	15.3	1.20 (1.12–1.29)
Others	11,633	189	162.5	12,767	188	147.3	1.10 (0.90–1.35)
α-glucosidase inhibitors	54,533	146	26.8	59,733	177	29.6	0.90 (0.73–1.12)

Combination drugs were included in each drug class by corresponding ingredients.

*AE* adverse event, *CI* confidence interval, *GLP-1RA* glucose like peptide-1 receptor analogues, *SGLT2 inhibitors* sodium-glucose co-transporter 2 inhibitors, *DPP4i* dipeptidyl peptidase 4 inhibitors.

^a^Reporting rate per 10,000 people was calculated over the total number of drug users of each class.

^b^Reporting ratio = reporting rate of women/ reporting rate of men.

The reporting rate of the individual event at the PT level with statistical significance and more than three times the difference between women and men was shown in Table 4. Majority of these events were dominated by women including headache in GLP1-RA users [risk ratio (95% confidence interval), 7.97 (1.01–62.78)], genital infection and urinary tract infections (all reported by women), in SGLT2i users, edema (fluid retention) [12.56 (2.91–54.13)] in TZD users, hyperglycemia [15.35 (8.54–27.60)] in insulin users, and urinary tract infection [5.66 (2.14–14.95)] in DPP-4 inhibitor users. The disparity of signal represented by ROR between genders generally aligned with the main results, reporting rate ratios (Table 4).

**Table 4:** Adverse event reports with substantial gender differences by antidiabetic subclass.

Adverse event (PT level)	Women			Men			Risk ratio^b^ (95% CI)
	Drug-AE pairs	Reporting rate^a^	ROR	Drug-AE pairs	Reporting rate^a^	ROR	
**GLP1-RA (Women = 3167; Men = 2800)**							
Headache	9	28.42	2.16	1	3.57	0.70	7.97 (1.01–62.78)
Vomiting	20	63.16	2.46	4	14.29	1.91	4.42 (1.51–12.92)
Dizziness	12	37.89	1.15	3	10.71	0.99	3.54 (1.01–12.52)
**SGLT2 inhibitor (Women = 72,400; Men = 78,767)**							
Urinary tract infection	19	2.62	18.63	0	0	–	–
Vaginitis	11	1.52	58.27	0	0	–	–
Genital infection	17	2.35	901.15	0	0	–	–
Pruritus, genital	44	6.08	430.56	1	0.13	259.37	47.87 (6.60–347.44)
Pruritus	20	0.97	2.76	2	0.25	0.19	10.88 (2.54–46.54)
Headache	11	1.03	1.52	2	0.25	0.44	5.98 (1.33–27.00)
**Thiazolidinedione (Women = 145,500; Men = 203.033)**							
Edema	18	1.24	11.57	2	0.10	2.61	12.56 (2.91–54.13)
Vomiting	7	0.48	0.70	1	0.05	0.17	9.77 (1.20–79.39)
Edema generalised	7	0.48	9.23	1	0.05	3.09	9.77 (1.20–79.39)
SGOT increased	7	0.48	7.84	2	0.10	1.62	4.88 (1.01–23.51)
SGPT increased	7	0.48	7.07	2	0.10	1.46	4.88 (1.01–23.51)
**Insulin (Women = 245,800; Men = 281,267)**							
Hyperglycemia	161	6.55	46.63	12	0.43	1.96	15.35 (8.54–27.60)
Pharyngitis	79	3.21	1.80	16	0.57	0.41	5.65 (3.30–9.67)
Injection site bruising	64	2.60	13.96	14	0.50	18.36	5.23 (29.3–9.33)
Sweating increased	52	2.12	2.41	15	0.53	0.77	3.97 (2.23–7.05)
Ketosis	18	0.73	241.42	6	0.21	33.10	3.43 (1.36–8.65)
Azotaemia	7	0.28	0.72	24	0.85	1.52	0.33 (0.14–0.77)
Hypertension	8	0.33	0.39	38	1.35	2.07	0.24 (0.11–0.52)
Pneumonia	7	0.28	0.25	39	1.39	0.85	0.21 (0.09–0.46)
Night sweats	0	–		20	0.71	46.98	–
**Sulfonylurea (Women = 660,133; Men = 823,033)**							
Granulocytopenia	20	0.30	0.85	5	0.06	0.21	5.05 (1.9–13.47)
Dyspepsia	55	0.83	1.77	17	0.20	0.75	4.09 (2.37–7.04)
Abdominal pain	29	0.44	1.62	11	0.13	0.66	3.33 (1.66–6.67)
Myalgia	15	0.23	1.35	6	0.07	0.74	3.16 (1.23–8.14)
Pneumonia	4	0.06	0.65	20	0.24	1.58	0.25 (0.09–0.74)
**Metformin (Women = 1,210,667, Men = 1,482,000)**							
Fracture	28	0.23	2.48	11	0.07	1.85	3.12 (1.55–6.26)
**DPP-4 inhibitor (Women = 812,667; Men = 1,045,533)**							
Urinary tract infection	22	0.27	6.79	5	0.05	3.45	5.66 (2.14–14.95)
Hyponatremia	13	0.16	11.28	3	0.03	0.90	5.58 (1.59–19.56)
Herpes zoster	11	0.14	5.60	3	0.03	0.96	4.72 (1.32–16.91)
Palpitation	14	0.17	1.15	4	0.04	0.68	4.50 (1.48–13.68)
Fracture	19	0.23	2.85	6	0.06	1.54	4.07 (1.63–10.20)
**AGI (Women = 54,533; Men = 59,733)**							
Diarrhea	2	0.37	0.60	11	1.84	2.11	0.20 (0.04–0.90)

*PT* preferred term, *ROR* reporting odds ratio, *CI* confidence interval.

^a^Reporting rate per 10,000 people was calculated over the total number of drug users of each class.

## Discussion

We investigated gender differences in the reporting of AE associated with the use of antidiabetic drugs by employing two different national databases, KAERS and the HIRA-NPS database. As a result, reporting of events following antidiabetic treatments was found to be predominant in women compared to men throughout the events categories and drug classes. 13 out of 17 SOCs levels with significant gender differences were reported more commonly by women than in men. The unequal reporting by gender were remarkable in gastrointestinal and metabolic and nutritional disorders considering both incidence rate and its gender discrepancy. By drug class, gender-specific reporting rates were observed in most antidiabetic classes, especially in newer oral antidiabetics such as GLP1-RA, SGLT2i, and TZD. Looking into PT level for each drug class, majorities of AEs with substantial disparity in reporting rate between genders were dominated by women, including urinary tract and genital infection after SGLT2i treatment, edema after TZD treatment, hyperglycemia in insulin users, and UTI in DPP-4i users.

Although quite a few studies explored gender differences in drug AEs, studies focused on antidiabetic drugs are sporadic and, to our knowledge, no studies have assessed the gender differences in reported AEs across entire antidiabetic agents at the national level in the real world situation. Admitting the different coverage of study drugs, our findings are consistent with those in the previous studies showing that the overall risk of adverse drug reporting is higher in women than men^7,13,18^. In a systematic analysis of AE reports gender differences in 20 treatment regimens, female patients were more likely to report drug-event combinations based on logarithmic reporting odds ratio^7^. A more recent observational study found that 322 out of 365 drug-event combinations with relevant gender differences were reported more often for women^8^.

Considering the magnitude of the gender difference and plausibility with antidiabetic medications, gender differences in gastrointestinal disorders reporting should be given attention. Gastrointestinal complaints such as diarrhea, nausea, and abdominal discomfort symptoms were common in metformin which is the first choice for diabetes with the highest volume of prescriptions. In a recent study using Dutch Pharmacovigilance data, female metformin users reported more common gastrointestinal side effects than men, with the greatest difference in nausea reporting rates (8.1% vs. 23.5%)^14^. GLP1-RA is another class strongly associated with the risk of gastrointestinal events, but whether it is gender-dependent is unknown^19^. While, a study of exenatide by using clinical trials data revealed that nausea, vomiting, and diarrhea were highly incident in females than males, in a randomized controlled trial, nausea incidence over 2 years in liraglutide treated diabetic patients was similar between the genders^20^. More frequent reporting by women for gastrointestinal events could be partly explained by the more prevalent functional gastrointestinal disorders in women. Also, it could attribute to a more sensitive attitude to a negative effect of diabetes on daily lives^21^. Metabolic and nutritional diseases were also reported more by women than men with high reporting frequencies posing clinical importance. Examining by drug class, much of the difference by gender appeared to be ascribed to some of the characteristic side effects such as genital and urinary tract infection, and edema, which were mainly resulted from the use of newer antidiabetic classes including GLP-1RA, SGLT2i, and TZD, and insulin therapy.

It is recognized that genital infection and urinary tract infection are more prevalent in women during treatment with SGLT2is^22^. Two population-based studies are in line with our results. A study in Singapore using national adverse reporting data identified majorities of reports describing urinary tract infection involved female patients^23^. A retrospective cohort study using claim data in the United States, the excess risk per 1000 people for genital infection in the SGLT2i group comparing to the DPP4i group was greater in women than in men (87.6 vs. 11.9)^18^. In animal studies, female rats preferentially use SGLT1 and SGLT2 transporters over other channels for Na^+^ reabsorption compared to male rats. While increased reliance on these glucose transporters in female animals is often linked to a higher incidence of glomerular hyperfiltration, whether these differences extend to humans is still unclear^24^. There also exists conflicting. In recent population-based cohort studies, gender differences were not significant in the incidence rate of genital infection in patients receiving SGLT2i, and overall safety profiles were not related to gender^25^. Indeed, the highest risk ratio was found in genital diseases, which may be related to the high prevalence of genital diseases in women. For example, diabetic women are more likely to develop vulvovaginal and pelvic infection^26,27^, and insulin deficiency followed by poor metabolic control results in reproductive dysfunction with decreased female hormones^28^.

It is accepted that edema can occur in patients treated with TZD. Edema itself may not be a serious side effect, but edema due to TZD may be a concern as it may be a sign of congestive heart disease^29^. Predominant reporting for edema in TZD treated women than men in our study was consistent with the previous study^30^. Female reproductive hormones such as estrogen are involved with the mechanisms to retain body water by increasing osmotic sensitivity, then women tend to experience fluid retention more common than men. However, it should be suspected that this may make it easier to overlook the edema as a precursor of coronary heart disease in women taking TZD.

Insulin mainly indicated for patients with severe hyperglycemia, not properly responding to oral hypoglycemic agents^31^. In addition to the high potential for adverse effects of insulin itself, the poor health status of their users can be attributed to the high reporting rate. A higher reporting rate of hyperglycemia in female insulin users is in line with previous studies which have indicated that women with diabetes have poorer glycemic control. Not presented in Table 4, hypoglycemia was also reported more by women than by men [RR (95% CI), 1.31 (1.17–1.47)], consistent with the results from the pooled analysis of clinical studies in which diabetic women experienced more hypoglycemic events, receiving higher weight-adjusted insulin doses than male^13^. More alleviated counter-regulatory responses to hypoglycemia in women was suggested as a mechanism^32,33^. Indeed, the fear of hypoglycemia has been described as a factor for suboptimal glycemic control in diabetic patients^34^.

Admitting that the AEs are mainly mild or modest and the reporting bias by gender can be inevitable, such a tendency of more frequently reported AEs by women throughout the antidiabetic drugs may be emphasizing efforts to improve compliance with the diabetic drug in women. Quite a few studies were showing that women are usually less likely to adhere to their medications compared with men^35^. A study using claim data presented that women were less likely than men to be adherent in medication for their chronic conditions including diabetes^36^. However, a French national survey study found no significant difference in adherence to diabetic treatment between the genders^37^.

To our knowledge, this study is the first to show the overview of the gender-related differences in reported AEs associated with entire antidiabetic drugs. We could also provide specific points of priority in the practice of diabetes treatment through analyzing the gender differences in the reporting rates at both macroscopic perspectives by body organs and at specific PT levels for each drug class. Very few studies have provided information on gender-specific AEs reporting in antidiabetic treatment making it difficult to fully compare with previous studies, but our results generally coincide with the previous studies: this implicitly supports the reliability of our research results and usefulness of the voluntary drug adverse report data in gender studies. This study had several methodological advantages: Based on two large-scale databases, we could complement the inherent limitation of voluntary drug AEs reporting data lack of denominator. Next, despite the inherent drawbacks of the KAERS database, our study has additional strengths in its generalizability by using a large size of nationwide data. According to a published guide for utilization of HIRA sample data, a test demonstrated that the estimation of diabetes prevalence and prescription of each hypoglycemic agent using the sample data aligned with the results of population analysis^38^.

Our study has several limitations. First, the ratio of reporting rates can not be a perfect approximate to the difference of actual risks by gender. Although there was a validation of such an assumption, it was in the case that the two compared drugs belong to the same therapeutic class and are used in similar conditions^39^. Gender have an impact on spontaneous reporting of AEs. A study indicated that healthcare professionals more frequently reported AEs for women. Conversely, serious reports were more frequently reported for men^40^, which was also supported by our findings. Another survey study showed the different attitudes for the disease between genders: Women dealt with the disease more seriously, and appealed to a higher impact on daily life and were more involved in self-management while men were dependent more on family support^41^. Second, different disease states such as comorbidities, diabetes severity, and age distribution according to gender affect the reporting rate. For instance, prevalence of morbidities such as UTI and genital infection were much higher in women than in men (Supplementary Table S1). Lastly, spontaneously reported AEs database has limitations such as possible under-reporting and absence of a causal relationship between reported drug and AE. The reporting rate in our study should not be viewed as a measure of AEs risk associated with antidiabetics. Further studies with an improved methodology to rule out the bias and confounding will be needed.

## Conclusion

The analysis of real-world data showed that reporting of AEs among diabetic drugs was more frequent in women than in men throughout the body organs and drug classes. This gender imbalance was pronounced in some of AEs specific to the newer antidiabetic classes such as headache in GLP1-RA, genital/urinary tract infection in SGLT2i, and edema in TZD. Gender differences in reported hyperglycemia among insulin users also greatly referred to women. Explanations for these different report levels by gender should be further explored.

## Supplementary information

Supplementary Table S1.

## Data Availability

KAERS and HIRA-NPS databases are available at the KIDS (https://open.drugsafe.or.kr/original/invitation.jsp) and the HIRA service webpage (https://opendata.hira.or.kr/op/opc/selectPatDataAplInfoView.do), respectively. The dataset used and/or analyzed during this study are not publicly available due to privacy or ethical restrictions.
