# Supplementary material for: Gender differences in adverse event reports associated with antidiabetic drugs
Source: Sci Rep. 2020 Oct 16;10:17545. doi: 10.1038/s41598-020-74000-4 (PMC7567832; doi:10.1038/s41598-020-74000-4)
Supplement: Supplementary file 1 — Supplementary Table S1. [file 41598_2020_74000_MOESM1_ESM.docx]

**Gender** **Differences in Adverse Event Reports Associated with Antidiabetic drugs**

Kyung-In Joung^1*^, Gyu-Won Jung^1*^, Han-Heui Park^1^, Hyesung Lee^1^, So-Hee Park^1^, Ju-Young Shin, PhD^1^

^1^School of Pharmacy, Sungkyunkwan University, Suwon, Korea

***Co-first authors**

**Corresponding author:** Ju-Young Shin, Ph.D.

School of Pharmacy, Sungkyunkwan University, 2066 Seobu-ro, Jangan-gu, Suwon, Gyeong gi-do, South Korea

Tel: 82-31-290-7702

Fax: 82-31-292-8800

E-mail: shin.jy@skku.edu

**Supplementary Table S1.** Demographic characteristics of diabetic population between January 1, 2016 and December 31, 2016.

| **Categories** | | **Women** | |  | **Men** | |  | **Difference in percentage** |
| --- | --- | --- | --- | --- | --- | --- | --- | --- |
|  |  | **1,798,633** | **%** |  | **2,036,300** | **%** |  |  |
| age | |  |  |  |  |  |  |  |
|  | ≤39 | 73,100 | (4.1) |  | 104,733 | (5.1) |  | -1.0 |
|  | 40-49 | 146,133 | (8.1) |  | 299,033 | (14.7) |  | -6.6 |
|  | 50-59 | 382,567 | (21.3) |  | 603,833 | (29.7) |  | -8.4 |
|  | 60-69 | 492,200 | (27.4) |  | 568,700 | (27.9) |  | -0.5 |
|  | 70-79 | 492,966 | (27.4) |  | 365,268 | (17.9) |  | 9.5 |
|  | ≥80 | 211,667 | (11.8) |  | 94,733 | (4.7) |  | 7.1 |
| Coexisting condition | |  |  |  |  |  |  |  |
|  | Cardiovascular disease | 481,700 | (26.8) |  | 515,633 | (25.3) |  | 1.5 |
|  | Hypertension | 998,633 | (55.5) |  | 1,054,167 | (51.8) |  | 3.7 |
|  | Dyslipidemia | 731,867 | (40.7) |  | 785,733 | (38.6) |  | 2.1 |
|  | Retinopathy | 403,900 | (22.5) |  | 382,767 | (18.8) |  | 3.7 |
|  | Neuropathy | 197,967 | (11.0) |  | 177,067 | (8.7) |  | 2.3 |
|  | Nephropathy | 67,000 | (3.7) |  | 99,867 | (4.9) |  | -1.2 |
|  | Peripheral vascular disease | 140,000 | (7.8) |  | 137,100 | (6.7) |  | 1.1 |
|  | Hypoglycemia | 9,733 | (0.5) |  | 9,467 | (0.5) |  | 0.0 |
|  | Genital infection | 98,533 | (5.5) |  | 5,433 | (0.3) |  | 5.2 |
|  | Urinary tract infection | 184,067 | (10.2) |  | 43,667 | (2.1) |  | 8.1 |
| Concomitant drug | |  |  |  |  |  |  |  |
|  | ACEi/ARBs | 454,966 | (25.3) |  | 536,100 | (26.3) |  | -1.0 |
|  | Calcium channel blockers | 486,033 | (27.0) |  | 468,533 | (23.0) |  | 4.0 |
|  | Diuretics | 316,000 | (17.6) |  | 247,500 | (12.2) |  | 5.4 |
|  | β-blockers | 312,800 | (17.4) |  | 330,600 | (16.2) |  | 1.2 |
|  | Statins | 976,667 | (54.3) |  | 939,900 | (46.2) |  | 8.1 |
|  | Antiplatelets | 697,800 | (38.8) |  | 793,333 | (39.0) |  | 0.2 |
|  | Anticoagulants | 123,900 | (6.9) |  | 145,800 | (7.2) |  | -0.3 |
| Antidiabetic drugs | |  |  |  |  |  |  |  |
|  | Insulin | 245,800 | (13.7) |  | 281,267 | (13.8) |  | -0.1 |
|  | Biguanide | 1,210,667 | (67.3) |  | ,482,000 | (72.8) |  | -5.5 |
|  | Sulfonylureas | 660,133 | (36.7) |  | 34,033 | (41) |  | -4.3 |
|  | α-glucosidase inhibitors | 54,533 | (3) |  | 59,733 | (2.9) |  | 0.1 |
|  | Thiazolidinediones | 145,500 | (8.1) |  | 203,033 | (10) |  | -1.9 |
|  | DPP-4 inhibitors | 812,667 | (45.2) |  | 1,045,533 | (51.3) |  | -6.2 |
|  | GLP-1RA | 3,167 | (0.2) |  | 2,800 | (0.1) |  | 0.0 |
|  | SGLT2 inhibitors | 72,400 | (4) |  | 78,767 | (3.9) |  | 0.2 |
|  | Others | 11,633 | (0.6) |  | 12,767 | (0.6) |  | 0.0 |
| Note: The number of population was extrapolated from the HIRA-NPS database by multiplying the identified numbers from HIRA-NPS by 100/3. | | | | | | | | |
| Abbreviations: ; HIRA-NPS, Health Insurance Review and Assessment Service-National Patients Sample; ARB, angiotensin 2 receptor blocker; ACEi, angiotensin converting enzyme inhibitorGLP-1RA, glucose like peptide-1 receptor analogues; SGLT2 inhibitors, sodium-glucose co-transporter 2 inhibitors; DPP-4 inhibitors, dipeptidyl peptidase 4 inhibitors | | | | | | | | |
